# Association of Telestroke Implementation with Onset-to-Admission Time and Clinical Outcomes in Patients with Acute Ischemic Stroke: A Retrospective Cohort Study in Bandung, Indonesia

**DOI:** 10.3390/healthcare14193174

**Published:** 2026-09-24

**Authors:** Andhira Riyan Ashori Hidayat, Lisda Amalia, Aih Cahyani

**Affiliations:** Department of Neurology, Faculty of Medicine, Padjadjaran University, Bandung 40161, Indonesia; dr.lisda@gmail.com (L.A.);

**Keywords:** Telestroke, acute ischemic stroke, telemedicine

## Abstract

**Background:** Telestroke may improve access to specialist stroke assessment in regions where neurological expertise is not immediately available. However, evidence regarding its implementation in low- and middle-income settings remains limited. This study explored the association between the Bandung Telestroke Activation System and onset-to-admission time and short-term clinical outcomes among patients with acute ischemic stroke. **Methods:** This retrospective cohort study included patients with acute ischemic stroke treated within the Bandung Telestroke referral network during the study period. Patients were categorized into Telestroke and Non-Telestroke groups according to whether their clinical management involved the Bandung Telestroke Activation System. The Telestroke group consisted of patients initially assessed at participating spoke hospitals and managed through remote neurological consultation before referral to Dr. Hasan Sadikin General Hospital, whereas the Non-Telestroke group consisted of patients who presented directly to the hub hospital without Telestroke support. Outcomes included onset-to-admission time, intravenous thrombolysis administration, and changes in National Institutes of Health Stroke Scale (NIHSS) scores during hospitalization. **Results:** The Telestroke group had a substantially shorter median onset-to-admission time than the Non-Telestroke group (3.5 vs. 20.0 h; *p* < 0.001) and a higher proportion of patients presenting within 4.5 h (59.0% vs. 8.3%; *p* < 0.001). After multivariable adjustment, Telestroke-supported care remained strongly associated with presentation within 4.5 h (aOR 11.20, 95% CI 4.16–30.11; *p* < 0.001). This association remained consistent after propensity score matching and residual adjustment (aOR 13.53, 95% CI 4.48–40.86; *p* < 0.001). Thrombolysis was more frequent in the Telestroke group in both the original and matched cohorts. Although the Telestroke group showed greater unadjusted ΔNIHSS, Telestroke was not independently associated with neurological improvement of ≥4 NIHSS points after multivariable adjustment (aOR 0.74, 95% CI 0.27–2.06; *p* = 0.564) or after propensity score matching (aOR 0.61, 95% CI 0.18–2.03; *p* = 0.419). **Conclusions:** The Bandung Telestroke Activation System was consistently associated with more timely hospital presentation and a higher proportion of thrombolysis administration. However, an independent association with early neurological improvement was not demonstrated after adjustment. These findings support the potential role of Telestroke in improving timely access to acute stroke care but should be interpreted cautiously given the retrospective design, different referral pathways, residual confounding, and limited sample size.

## 1. Introduction

Telestroke refers to the application of telemedicine systems to support the remote assessment and management of patients with acute stroke, particularly when timely access to stroke specialists is limited. Telestroke can support clinical decision-making regarding emergency stroke management, including the administration of intravenous thrombolysis with tissue plasminogen activator (IV tPA) [1,2]. Telestroke aims to improve access to specialized stroke care for patients who experience delays in accessing appropriate stroke services at their initial healthcare facilities. Previous studies have reported that telestroke networks are associated with improved access to specialist consultation and increased use of intravenous thrombolysis in patients with acute ischemic stroke [3].

According to the World Health Organization (WHO), stroke was the second leading cause of death worldwide in 2017 [4]. According to Indonesian Basic Health Research (Riskesdas), the prevalence of stroke increased from 7 to 10.9 per 1000 population between 2003 and 2018 [5].

A total of 141 ischemic stroke patients received medical care at Dr. Hasan Sadikin General Hospital in 2021. Among these, 118 patients (83.69%) were classified as “late,” while 23 patients (16.31%) were categorized as “not late” [6]. The time from symptom onset to hospital presentation is an important factor in acute stroke management. In patients with acute ischemic stroke, intravenous thrombolysis with recombinant tissue plasminogen activator (rtPA) is an established reperfusion treatment for eligible patients presenting within the appropriate therapeutic window [7]. Intravenous thrombolysis is recommended in appropriate eligible patients by major stroke guidelines, including those from the American Heart Association/American Stroke Association (AHA/ASA) and the European Stroke Organisation (ESO) [8,9].

Timely intervention is an important determinant of outcomes in acute ischemic stroke. Insufficient knowledge regarding stroke symptoms, delays in hospital arrival, failure to recognize stroke as an emergency, financial constraints, and limited access to referral pathways for thrombolytic therapy are among the factors contributing to delays in the management of acute ischemic stroke [6]. A major barrier to timely intravenous thrombolysis is delayed access to hospitals capable of providing appropriate stroke evaluation and reperfusion therapy. Comprehensive stroke care, from acute treatment to neurorehabilitation, is supported by facilities with appropriate stroke care capacity, including Comprehensive Stroke Centers. A Comprehensive Stroke Center is defined as a facility or system equipped with personnel, infrastructure, expertise, and programs necessary to diagnose and treat stroke patients requiring intensive medical and surgical care, specialized tests, or interventional therapy [10,11]. To optimize service delivery, strategies are needed to improve timely access to specialist stroke assessment and reperfusion therapy, particularly in large geographical areas where access to comprehensive stroke services remains limited.

Telestroke may provide a practical approach to improving access to specialist assessment and supporting clinical decision-making for patients with acute ischemic stroke, particularly when intravenous thrombolysis is being considered. Previous studies have suggested that telestroke networks may help reduce delays and improve access to time-sensitive stroke treatment [12]. Depending on local infrastructure and treatment capacity, different hub-and-spoke models may be used to facilitate stroke care and interhospital referral. The organization of patient transfer and thrombolysis administration should be adapted to the capabilities of the participating hospitals and the regional stroke care pathway.

## 2. Methods

### 2.1. Research Design

This retrospective cohort study was conducted to evaluate the association of the Bandung Telestroke Activation System with treatment timelines and short-term clinical outcomes among patients with acute ischemic stroke. The study was conducted within the Bandung stroke referral network, with Dr. Hasan Sadikin General Hospital serving as the comprehensive stroke center (hub) and five referral hospitals—Ujung Berung Regional General Hospital, Bandung Kiwari Regional General Hospital, Sartika Asih Bhayangkara Level II Hospital, dr. M. Salamun Air Force Level II Hospital, and Bandung Advent Hospital—serving as spoke hospitals (Table 1; Figure 1). These hospitals were selected based on their geographic proximity to the hub, established stroke care capacity, and readiness to implement the Bandung Telestroke Activation System, thereby providing the infrastructure for coordinated neurological consultation, clinical communication, and interhospital referral.

The Bandung Telestroke Activation System uses a web-based platform to facilitate real-time communication between spoke hospitals and the hub hospital. At the spoke hospitals, designated healthcare personnel enter relevant clinical information, including the patient’s medical history, symptom onset time, neurological examination findings, supporting investigations, and National Institutes of Health Stroke Scale (NIHSS) score, into the Telestroke platform. The submitted information is recorded in the system and generates a real-time notification to the hub hospital. The hub team then reviews the available clinical information and provides feedback to the referring spoke hospital, with a target response time of less than 10 min. Based on the clinical assessment and available information, the patient may be referred to the hub hospital for further management or continue treatment at the spoke hospital. This workflow was designed to support timely communication and clinical decision-making across participating hospitals (Figure 2). This study included consecutive eligible patients treated between July 2024 and January 2025. As this was a retrospective study, the research was based exclusively on existing medical records. All data access, extraction, and analysis were performed only after approval had been granted by the Research Ethics Committee of Universitas Padjadjaran (Approval No. 809/UN6.KEP/EC/2024; approved on 17 July 2024). Thus, the study period reflects the timeframe during which patients received clinical care, whereas data collection for research purposes commenced only after ethical approval had been obtained.

### 2.2. Research Subject

The study population consisted exclusively of patients with acute ischemic stroke who met the predefined eligibility criteria. Patients with hemorrhagic stroke were not included. The time from symptom onset to hospital arrival varied among patients, and not all patients presented within the therapeutic window for intravenous thrombolysis. The proportion of patients presenting within 4.5 h of symptom onset was reported for each group to describe timely presentation within the conventional reperfusion window. Intravenous thrombolysis administration was assessed as a secondary clinical outcome based on documented treatment in the medical records. Because detailed thrombolysis eligibility criteria were not consistently available in the retrospective records, treatment rates among confirmed eligible patients could not be reliably calculated. These patients were divided into two groups based on their Telestroke status:

1. Telestroke Group: Patients who initially presented to one of the five participating spoke hospitals and were managed through the Bandung Telestroke Activation System. Following remote neurological consultation and clinical decision-making, these patients were referred to Dr. Hasan Sadikin General Hospital (RSHS) for further management.
2. Non-Telestroke group: Patients with acute ischemic stroke who presented directly to RSHS and received conventional care without activation of the Telestroke system. Group allocation was therefore determined by the clinical presentation and referral pathway during routine care rather than by investigator assignment.

Both groups were identified during the same study period, from July 2024 to January 2025. A total of 267 patients were included in the final analysis, comprising 39 patients in the Telestroke group and 228 patients in the Non-Telestroke group (Figure 3).

### 2.3. Inclusion and Exclusion Criteria

Inclusion Criteria:

- Patients with a diagnosis of acute ischemic stroke based on clinical assessment and supporting examinations.
- Age > 18 years old.
- Complete medical record data, including time of symptom onset, time of hospital arrival, assessment using the National Institutes of Health Stroke Scale (NIHSS), and clinical outcomes.

Exclusion Criteria:

- Incomplete or uninterpretable medical records.
- Patients with a diagnosis of non-stroke or other neurological conditions that resemble a stroke.

### 2.4. Outcome Variables

The primary outcome was onset-to-admission time, defined as the time interval in hours between symptom onset and hospital arrival and used to describe the timeliness of access to hospital-based stroke care rather than diagnostic performance.

Clinical outcomes included documented intravenous thrombolysis administration and neurological status assessed using the National Institutes of Health Stroke Scale (NIHSS). NIHSS was recorded at admission and discharge, and the change in NIHSS (ΔNIHSS) was calculated as the difference between admission and discharge NIHSS scores. Intravenous thrombolysis administration was analyzed based on treatment documented in the medical records rather than confirmed eligibility for reperfusion therapy.

Demographic characteristics and vascular risk factors were also collected, including age, sex, hypertension, diabetes mellitus, dyslipidemia, previous stroke, atrial fibrillation, and other relevant comorbidities.

### 2.5. Data Collection Method

Data were obtained retrospectively from existing clinical and medical records of eligible patients with acute ischemic stroke treated between July 2024 and January 2025. The collected data included demographic characteristics, symptom onset time, hospital arrival time, relevant medical history, neurological examination findings, therapeutic interventions, including intravenous thrombolysis, and clinical outcomes at hospital discharge. All data were extracted retrospectively from records generated during routine clinical care.

### 2.6. Statistical Analysis

Statistical analyses were performed using IBM SPSS Statistics for Windows, Version 30.0 (IBM Corp., Armonk, NY, USA). Continuous variables were assessed for normality using the Shapiro–Wilk test. Normally distributed variables are presented as mean ± standard deviation (SD) and compared using the independent-samples *t*-test, whereas non-normally distributed variables are presented as median and interquartile range (IQR) and compared using the Mann–Whitney U test. Categorical variables are presented as frequencies and percentages and compared using the chi-square or Fisher’s exact test, as appropriate. All tests were two-sided, with *p* < 0.05 considered statistically significant. Between-group differences were reported with effect estimates and 95% confidence intervals (CIs). The association between Telestroke use and intravenous thrombolysis was expressed as a crude odds ratio (OR) with a 95% confidence interval (CI). Potential confounding and selection bias were considered when interpreting the findings because of differences in baseline characteristics between groups. Missing data were assessed before analysis, and available observations were used without imputation of missing clinical outcomes. Given the small Telestroke sample and limited number of thrombolysis events, adjusted and sensitivity analyses were considered cautiously to avoid unstable estimates.

Multivariable logistic regression was performed to evaluate the independent association between Telestroke-supported care and two prespecified binary outcomes: presentation within 4.5 h of symptom onset and neurological improvement defined as a reduction in NIHSS score of ≥4 points. The regression models included Telestroke status, age, sex, baseline NIHSS, hypertension, diabetes mellitus, smoking status, previous stroke, dyslipidemia, atrial fibrillation, and chronic kidney disease. Adjusted odds ratios (aORs) with 95% confidence intervals (CIs) were reported. Model fit was assessed using the Omnibus test, Nagelkerke R^2^, and the Hosmer–Lemeshow goodness-of-fit test.

As a sensitivity analysis, propensity score matching (PSM) was performed to reduce measured baseline differences between the Telestroke and Non-Telestroke groups. Propensity scores were estimated using age, sex, baseline NIHSS, hypertension, diabetes mellitus, smoking status, previous stroke, dyslipidemia, atrial fibrillation, and chronic kidney disease. Patients were matched without replacement using a maximum 1:3 matching ratio and a caliper of 0.370, corresponding to 0.20 standard deviations of the logit of the propensity score. Covariate balance after matching was assessed using standardized mean differences (SMDs). An absolute SMD of <0.10 was considered indicative of good balance.

The matched cohort was subsequently compared with respect to presentation within 4.5 h, intravenous thrombolysis, ΔNIHSS, neurological improvement of ≥4 NIHSS points, and length of hospital stay. Additional logistic regression models were performed within the matched cohort to assess whether the associations between Telestroke and the prespecified binary outcomes remained after residual adjustment for age and baseline NIHSS. Because of the relatively small Telestroke sample and limited number of outcome events, the adjusted and propensity score analyses were interpreted as supportive or sensitivity analyses rather than as evidence of causality.

### 2.7. Ethical Considerations

The study was conducted in accordance with the principles of the Declaration of Helsinki and was approved by the Research Ethics Committee of Universitas Padjadjaran, Bandung, Indonesia (Approval No. 809/UN6.KEP/EC/2024; approved on 17 July 2024). This retrospective cohort study included eligible patients with acute ischemic stroke treated during the Bandung Telestroke Activation pilot project from July 2024 to January 2025. The study used clinical records generated during routine patient care. Following ethical approval, eligible records from the pilot project period were retrospectively accessed, extracted, and analyzed for research purposes. No research related data collection was performed before ethical approval was obtained.

## 3. Result

### 3.1. Demographic Characteristics

Between July 2024 and January 2025, a total of 267 acute stroke patients were analyzed in this study. Of these, 39 patients (14.6%) were managed with the support of the Telestroke system, while 228 patients (85.4%) were managed through the Non-Telestroke pathway without Telestroke consultation.

The Telestroke and Non-Telestroke groups had similar age and sex distributions. The mean age was 57.41 ± 13.21 years in the Telestroke group and 57.64 ± 13.21 years in the Non-Telestroke group, with no statistically significant difference (*p* = 0.918). Similarly, the proportion of male patients did not differ significantly between the groups (61.5% vs. 50.9%; *p* = 0.218). The mean age observed in this cohort was consistent with the relatively younger age at stroke onset reported in Asian populations compared with Western populations [4]. However, these similarities in age and sex should not be interpreted as indicating overall baseline equivalence, as differences were observed in several vascular and clinical characteristics between the groups.

The distribution of vascular risk factors showed significant differences in several key comorbidities. Hypertension was more common in the Non-Telestroke group than in the Telestroke group (91.7% vs. 79.5%; *p* = 0.038). In addition, the prevalence of dyslipidemia (70.2% vs. 25.6%; *p* < 0.001) and previous stroke history (43.0% vs. 7.7%; *p* < 0.001) were also significantly higher in the Non-Telestroke group. This is in line with the study of Feigin et al., where hypertension and dyslipidemia are the largest risk factors (more than 80% in ischemic stroke patients) for stroke burden globally [4]. Another finding from O’Donnell et al., through the INTERSTROKE study that covered 32 countries on different continents, including the Asian region. In the study, more than 90% of stroke incidence was caused by modifiable risk factors, with hypertension and dyslipidemia as the main risk factors [13] (Table 2).

### 3.2. Onset-to-Admission Time

The onset-to-admission interval was used to describe the time from symptom onset to hospital arrival and the timeliness of access to hospital-based stroke care. The Telestroke group showed a significantly shorter onset-to-admission time, with a median of 3.5 h (IQR 2.5–6.0), compared with 20.0 h (IQR 7.0–72.0) in the Non-Telestroke group (*p* < 0.001). This measure reflects differences in prehospital delay and access to hospital care rather than the speed or accuracy of stroke diagnosis. The observed difference should therefore be interpreted cautiously, particularly because patients in the Telestroke and Non-Telestroke groups followed different pathways to the hub hospital. Timely access to hospital-based stroke care remains an important component of effective stroke systems. Network-based systems of care may integrate prehospital notification, stroke code activation, interhospital communication, and telemedicine to support coordinated stroke management [14,15]. However, the present study did not directly measure diagnostic time or reperfusion-related treatment intervals.

### 3.3. Neurological Severity

The severity of neurological impairment at hospital admission was assessed using the National Institutes of Health Stroke Scale (NIHSS). The median admission NIHSS score was 7 (IQR, 4–13) in the Telestroke group and 7 (IQR, 4–11) in the Non-Telestroke group, with no statistically significant difference between the groups (*p* = 0.478) (Table 3). These findings indicate that the groups had a similar level of neurological impairment at the time of hospital admission. The distribution of onset-to-admission time and changes in NIHSS between the two groups is presented in (Figure 4).

Although admission NIHSS was comparable between the groups, it was retained as an important covariate in subsequent multivariable and propensity score analyses to account for potential differences in baseline neurological severity. This approach allowed the association between Telestroke management and clinical outcomes to be assessed while accounting for differences in initial stroke severity.

### 3.4. Neurological Status at Discharge

The NIHSS score at discharge did not differ significantly between the Telestroke and Non-Telestroke groups, with a median of 4 (IQR 2–9) in the Telestroke group and 4 (IQR 3–7) in the Non-Telestroke group (*p* = 0.652) (Table 3). Although discharge NIHSS provides an indication of neurological status at the end of hospitalization, it represents a single time point and may not fully capture change in neurological status during hospitalization. Changes in NIHSS scores from admission to discharge (ΔNIHSS) may therefore provide additional information regarding early neurological improvement. However, given the retrospective design of this study, potential baseline differences between the groups, and the absence of long-term functional outcomes, differences in ΔNIHSS should be interpreted cautiously and cannot be considered evidence of long-term clinical benefit.

### 3.5. Neurological Improvement

Changes in NIHSS scores from admission to discharge were assessed using ΔNIHSS, calculated as admission NIHSS minus discharge NIHSS. The Telestroke group had a higher median ΔNIHSS than the Non-Telestroke group (3 [IQR, 2–5] vs. 1 [IQR, 0–4]; *p* < 0.001) (Table 3). The distribution of ΔNIHSS between the two groups is shown in Figure 4.

The proportion of patients with an NIHSS improvement of ≥4 points was 35.9% in the Telestroke group and 28.5% in the Non-Telestroke group, with no significant difference between the groups (*p* = 0.350). After adjustment for potential confounders, Telestroke was not significantly associated with NIHSS improvement of ≥4 points (aOR, 0.74; 95% CI, 0.27–2.06; *p* = 0.564). This finding remained consistent after propensity score matching and residual adjustment (aOR, 0.61; 95% CI, 0.18–2.03; *p* = 0.419).

This suggests that the higher unadjusted ΔNIHSS observed in the Telestroke group was not independently associated with Telestroke management after adjustment.

### 3.6. Thrombolysis Administration

Intravenous thrombolysis was administered to 8 patients (20.5%) in the Telestroke group and 6 patients (2.6%) in the Non-Telestroke group (*p* < 0.001). The crude odds of receiving intravenous thrombolysis were higher in the Telestroke group (OR, 9.55; 95% CI, 3.11–29.36). Because detailed thrombolysis eligibility criteria were not consistently available in the retrospective records, these findings reflect documented thrombolysis administration in the study cohort rather than treatment rates among confirmed eligible patients. Therefore, the difference should be interpreted as an association and not as evidence that Telestroke increased the use of thrombolysis among eligible patients.

### 3.7. Multivariable Analysis

Multivariable logistic regression analyses were performed to determine whether the observed associations between Telestroke management and clinical outcomes remained after adjustment for potential confounding factors. Two clinically relevant outcomes were examined: presentation within 4.5 h of symptom onset and neurological improvement defined as a reduction in the NIHSS score of ≥4 points. The models included Telestroke status, age, sex, admission NIHSS, hypertension, diabetes mellitus, smoking, previous stroke, dyslipidemia, atrial fibrillation, and chronic kidney disease. For presentation within 4.5 h of symptom onset, Telestroke management was independently associated with a substantially higher likelihood of early hospital presentation (adjusted odds ratio [aOR], 11.20; 95% confidence interval [CI], 4.16–30.11; *p* < 0.001). Previous stroke was also independently associated with presentation within 4.5 h (aOR, 5.18; 95% CI, 1.61–16.71; *p* = 0.006). None of the other covariates showed a statistically significant association with this outcome (Table 3). The model was statistically significant overall (Omnibus *p* < 0.001), explained 37.6% of the variance according to the Nagelkerke R^2^, and demonstrated adequate goodness of fit (Hosmer–Lemeshow *p* = 0.740) (Table 4).

For neurological improvement, Telestroke status was not independently associated with achieving an NIHSS reduction of ≥4 points after adjustment (aOR, 0.74; 95% CI, 0.27–2.06; *p* = 0.564). In contrast, higher-admission NIHSS was strongly associated with greater odds of achieving this degree of improvement (aOR, 1.35; 95% CI, 1.25–1.46; *p* < 0.001). Previous stroke was also independently associated with the outcome (aOR, 2.59; 95% CI, 1.21–5.54; *p* = 0.014). The remaining covariates were not significantly associated with neurological improvement (Table 5). The overall model was statistically significant (Omnibus *p* < 0.001), with a Nagelkerke R^2^ of 0.403 and adequate goodness of fit according to the Hosmer–Lemeshow test (*p* = 0.274).

Taken together, the multivariable analysis demonstrated a consistent association between Telestroke management and presentation within 4.5 h of symptom onset. However, the greater change in NIHSS observed in the unadjusted analysis was not reflected in a significantly higher likelihood of achieving an NIHSS improvement of ≥4 points after adjustment. These findings were further examined in a propensity score matching sensitivity analysis.

### 3.8. Propensity Score Matching Sensitivity Analysis

Propensity score matching (PSM) was performed as a sensitivity analysis to assess the robustness of the observed associations after accounting for differences in baseline characteristics between the Telestroke and Non-Telestroke groups. The propensity score was estimated using age, sex, admission NIHSS, hypertension, diabetes mellitus, smoking, previous stroke, dyslipidemia, atrial fibrillation, and chronic kidney disease. Matching was performed without replacement, with a maximum matching ratio of 1:3 and a caliper of 0.370 on the logit propensity score scale. Among the 39 patients in the Telestroke group, 28 (71.8%) were successfully matched with at least one Non-Telestroke control, whereas 11 (28.2%) had no eligible match within the predefined caliper. Of the matched Telestroke patients, 22 were matched with three controls, 2 with two controls, and 4 with one control, resulting in 74 selected Non-Telestroke controls. The final matched cohort therefore comprised 102 patients, including 28 Telestroke patients and 74 Non-Telestroke controls (Table 6).

After matching, the baseline characteristics were generally more comparable between the two groups. Good balance was achieved for age, hypertension, diabetes mellitus, smoking, previous stroke, and chronic kidney disease. However, some residual imbalance remained for admission NIHSS, sex, dyslipidemia, and atrial fibrillation, as reflected by standardized mean differences (SMDs) exceeding 0.10 for these variables (Table 7).

In the matched cohort, the proportion of patients presenting within 4.5 h of symptom onset remained significantly higher in the Telestroke group than in the Non-Telestroke group (57.1% vs. 9.5%; *p* < 0.001). Thrombolysis administration was also more frequent among Telestroke patients (17.9% vs. 4.1%; *p* = 0.034). In contrast, no significant differences were observed in ΔNIHSS (*p* = 0.303), the proportion of patients achieving an NIHSS improvement of ≥4 points (25.0% vs. 37.8%; *p* = 0.223), or length of hospital stay (*p* = 0.163) (Table 8).

To further account for the residual differences observed after matching, logistic regression models were performed with additional adjustment for age and admission NIHSS. In the matched cohort, Telestroke remained strongly associated with presentation within 4.5 h of symptom onset (aOR 13.53, 95% CI 4.48–40.86; *p* < 0.001). In contrast, Telestroke was not significantly associated with achieving an NIHSS improvement of ≥4 points (aOR 0.61, 95% CI 0.18–2.03; *p* = 0.419), whereas higher-admission NIHSS was independently associated with greater odds of achieving this outcome (aOR 1.39, 95% CI 1.22–1.59; *p* < 0.001) (Table 9).

## 4. Discussion

### 4.1. Principal Findings

This study found that Telestroke-supported care was associated with earlier hospital presentation in patients with acute ischemic stroke. The median onset-to-admission time was shorter in the Telestroke group than in the Non-Telestroke group (3.5 vs. 20.0 h), and more patients presented within 4.5 h (59.0% vs. 8.3%). This association remained significant after multivariable adjustment and propensity score matching.

Telestroke was also associated with a higher proportion of documented intravenous thrombolysis. This finding is consistent with previous studies showing that telestroke can improve access to specialist stroke assessment and support thrombolysis decisions [3,12]. However, detailed thrombolysis eligibility was not consistently available in our records, so this result should be interpreted as documented treatment administration rather than treatment among confirmed eligible patients.

Although ΔNIHSS was greater in the Telestroke group in the unadjusted analysis, Telestroke was not independently associated with an NIHSS improvement of ≥4 points after adjustment. Overall, the observed association was more closely related to timely access to hospital-based stroke care than to an independent association with short-term neurological improvement.

### 4.2. Timely Hospital Presentation

The large difference in onset-to-admission time is an important finding of this study. Telestroke may help create a clearer pathway between spoke hospitals and the hub hospital, allowing patients with suspected acute stroke to receive specialist input and appropriate referral more efficiently [12,14,15].

However, onset-to-admission time is also influenced by factors before hospital arrival, such as symptom recognition, healthcare-seeking behavior, transportation, and access to emergency services. These factors were not available in our dataset. Therefore, the shorter onset-to-admission time should not be interpreted as evidence that Telestroke directly reduced prehospital delay. Rather, it indicates an association between the Telestroke pathway and more timely access to hospital-based stroke care.

### 4.3. Intravenous Thrombolysis

The proportion of patients receiving documented intravenous thrombolysis was higher in the Telestroke group than in the Non-Telestroke group (20.5% vs. 2.6%). A similar difference remained after propensity score matching (17.9% vs. 4.1%). This finding is consistent with previous evidence that telestroke can facilitate specialist assessment and access to reperfusion treatment [3,12]. Previous work has also examined biological changes associated with thrombolytic treatment in acute ischemic stroke [16,17].

Nevertheless, the result should be interpreted cautiously because the available records did not allow us to determine thrombolysis eligibility consistently. In addition, differences in presentation time and referral pathways may have contributed to the higher thrombolysis rate. Therefore, the observed difference represents an association and cannot be attributed solely to Telestroke.

### 4.4. Neurological Outcomes

The Telestroke group had a greater median ΔNIHSS than the Non-Telestroke group (3 vs. 1; *p* < 0.001). However, this difference was not maintained after adjustment. Telestroke was not significantly associated with an NIHSS improvement of ≥4 points in either the multivariable analysis or the propensity-matched analysis.

This finding suggests that the higher unadjusted ΔNIHSS may have been influenced by other patient or treatment characteristics. In contrast, admission NIHSS remained strongly associated with neurological improvement. Therefore, baseline stroke severity appears to be an important factor when interpreting early changes in NIHSS.

### 4.5. Clinical and Health-System Implications

The findings support the potential role of Telestroke as part of a regional stroke system of care. Telestroke may be particularly useful for hospitals that do not have immediate access to neurologists or comprehensive stroke services [14,15]. For the Bandung network, future evaluation should focus on process measures that are more directly related to Telestroke performance, including door-to-consultation, door-to-imaging, door-to-needle, and interhospital transfer times. These measures would provide a clearer assessment of how the system affects acute stroke management.

### 4.6. Limitations

This study has several limitations. First, the retrospective observational design limits the ability to establish a causal relationship between Telestroke implementation and clinical outcomes. Although multivariable logistic regression and propensity score matching were performed, residual confounding from unmeasured factors cannot be excluded. The two groups also followed different referral pathways, which may have contributed to differences in patient characteristics and access to care.

Second, propensity score matching improved the comparability between groups, but complete balance was not achieved. Only 28 of 39 Telestroke patients were successfully matched, while 11 patients remained unmatched. Some residual imbalance was also present in admission NIHSS, sex, dyslipidemia, and atrial fibrillation. Therefore, the post-matching findings should be interpreted as sensitivity analyses rather than definitive evidence of a causal effect.

Third, onset-to-admission time is influenced by factors occurring before hospital arrival, including symptom recognition, healthcare-seeking behavior, transportation, and access to emergency services. These factors were not systematically available in the retrospective dataset. Future prospective studies should therefore include standardized process measures, such as door-to-consultation, door-to-imaging, door-to-needle, and interhospital transfer times.

Fourth, neurological outcomes were assessed using in-hospital NIHSS measures. The study did not include long-term functional outcomes, such as the 90-day modified Rankin Scale (mRS). Therefore, changes in NIHSS during hospitalization should not be interpreted as evidence of long-term functional benefit.

Finally, the relatively small Telestroke sample and the single regional referral network may limit the precision and generalizability of the findings. Further multicenter prospective studies with larger populations and standardized long-term outcomes are needed to confirm these findings.

## 5. Conclusions

In this retrospective cohort study, management through the Bandung Telestroke Activation System was consistently associated with more timely hospital presentation among patients with acute ischemic stroke. The association remained present after adjustment for measured baseline differences using multivariable adjustment and propensity score matching. The Telestroke group also had a higher proportion of patients receiving intravenous thrombolysis, including after propensity score matching.

However, the greater unadjusted ΔNIHSS observed in the Telestroke group was not maintained after propensity score matching, and Telestroke-supported care was not independently associated with achieving a ≥4-point reduction in NIHSS. Therefore, the findings provide stronger evidence for an association with timely access to hospital-based stroke care than for an independent effect on early neurological recovery. Given the retrospective design, different referral pathways, residual confounding, and limited sample size, these findings should be considered exploratory and hypothesis-generating. Prospective studies with standardized reperfusion eligibility assessment, larger multicenter populations, and long-term functional outcomes are warranted to determine the clinical effectiveness and broader health-system impact of Telestroke implementation.

## Figures and Tables

**Figure 1:**
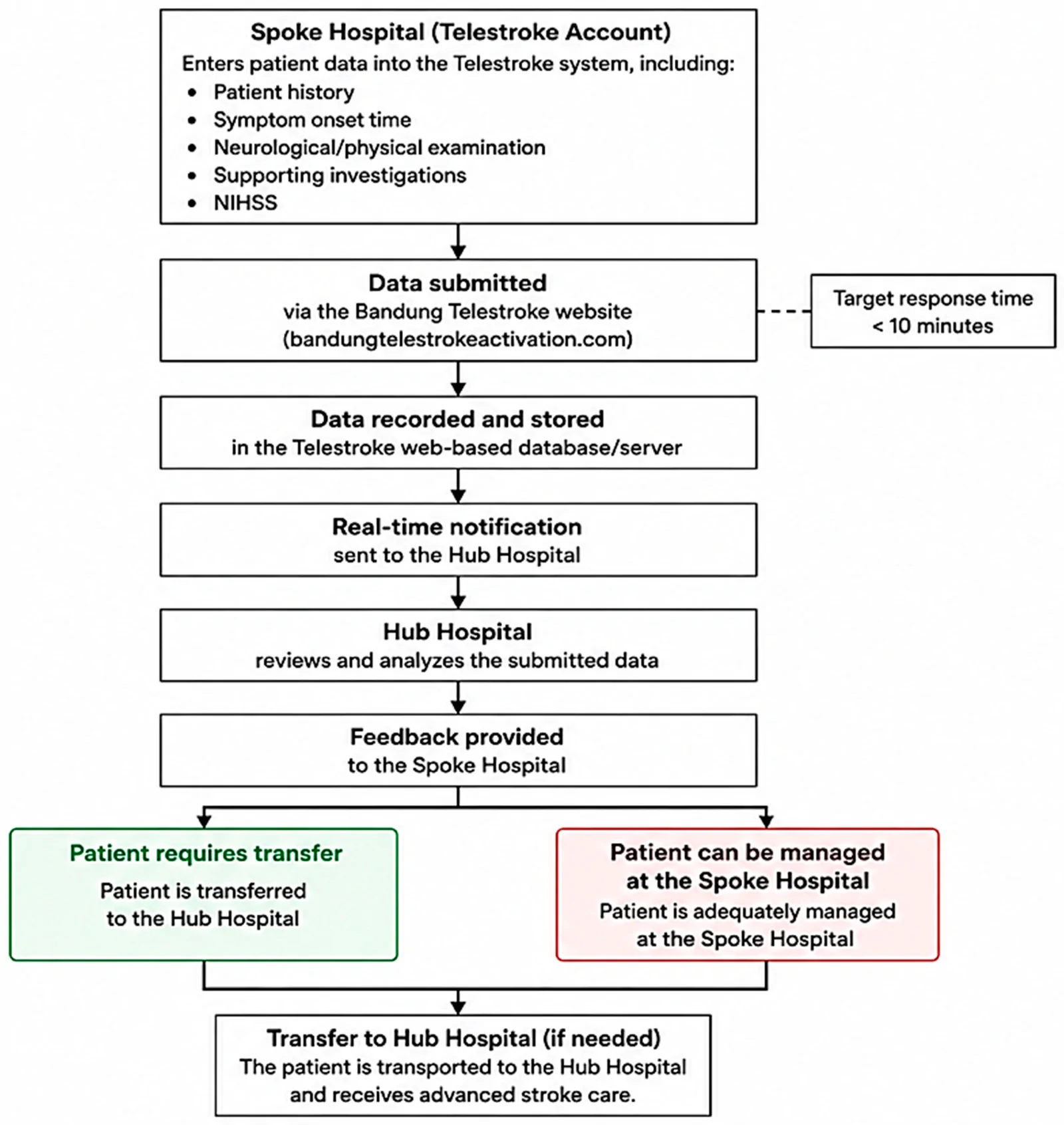
Workflow of the Bandung Telestroke Activation System.

**Figure 2:**
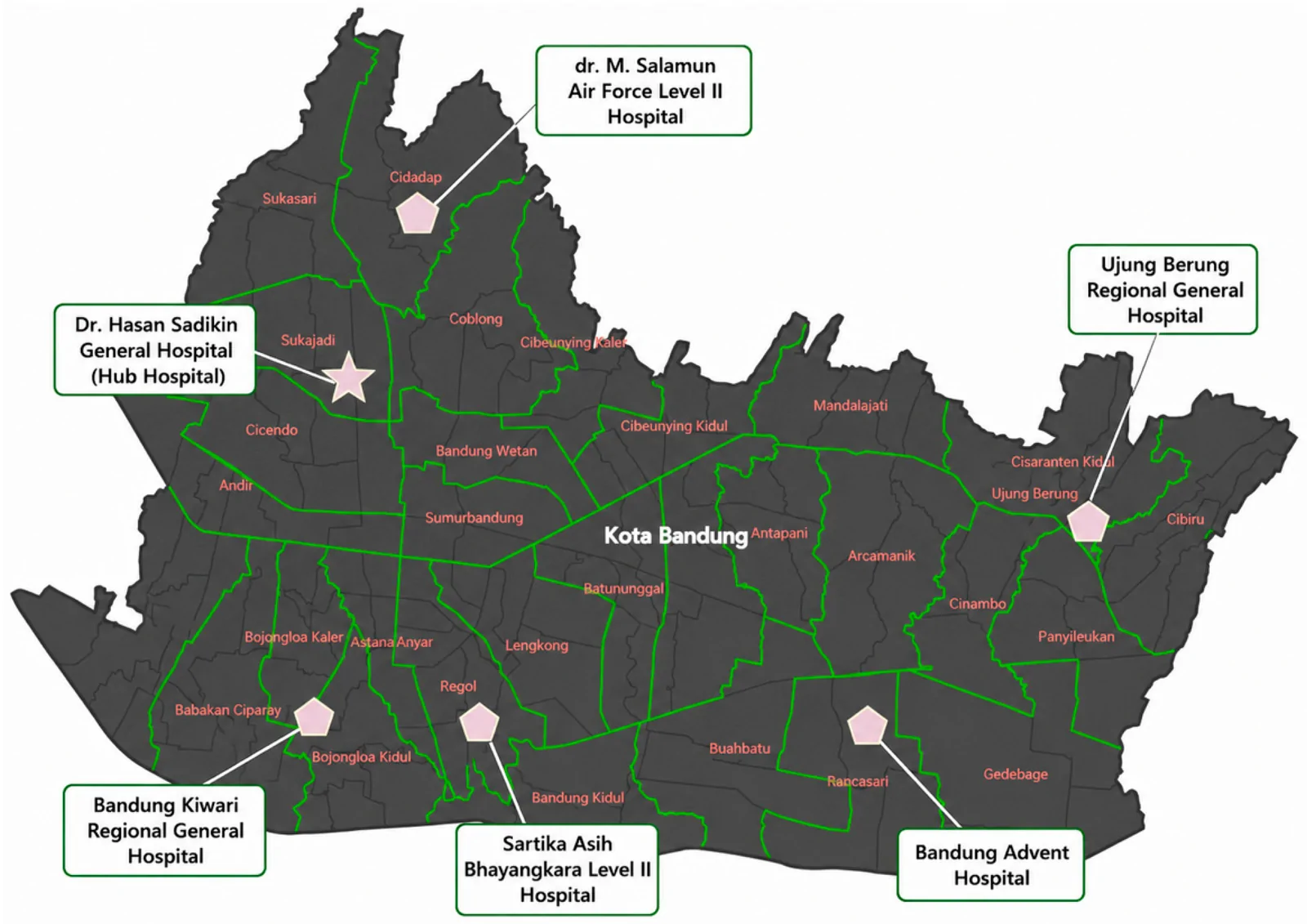
Map of spoke hospitals and their position against hub hospitals.

**Figure 3:**
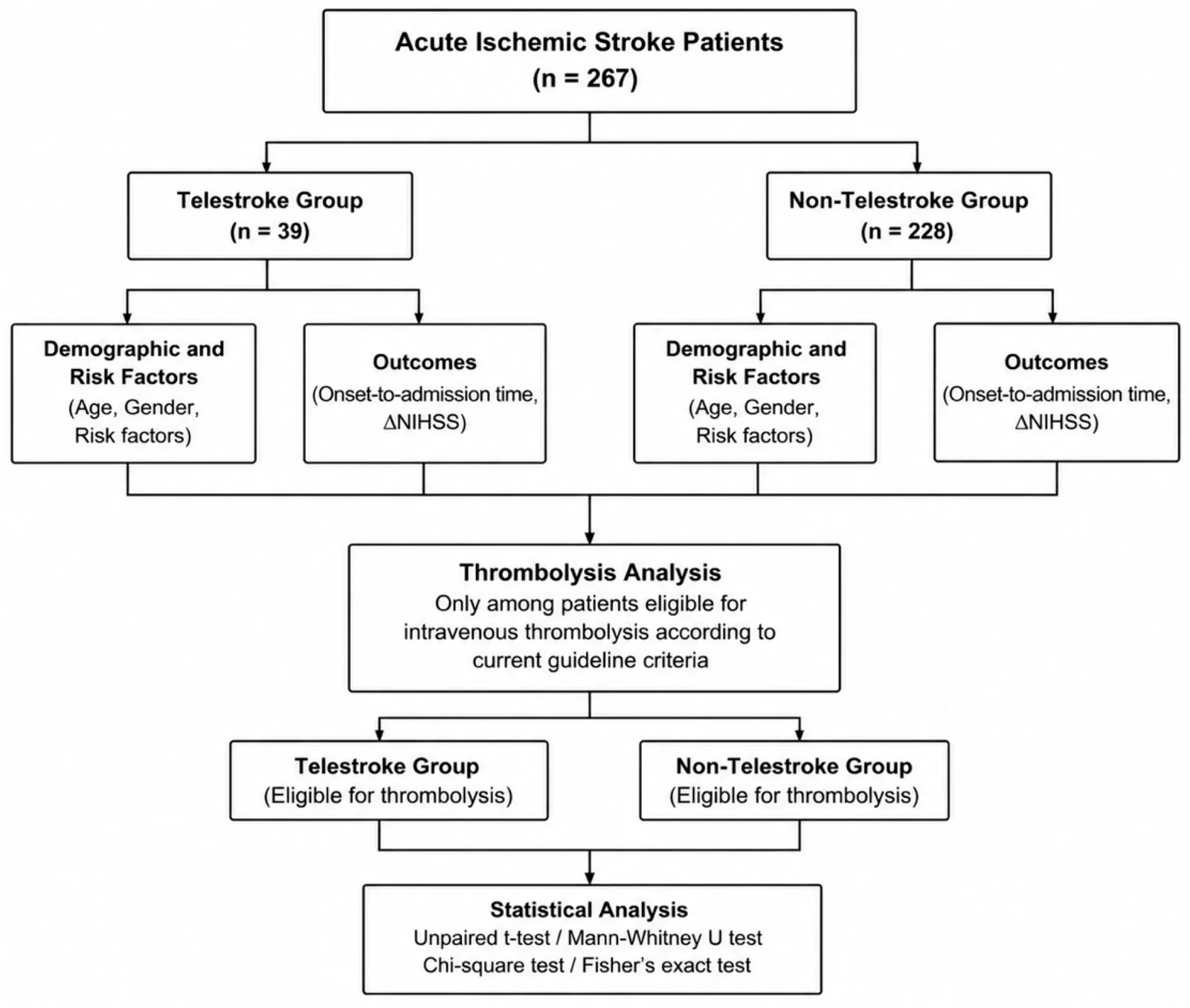
Flow of research subject recruitment.

**Figure 4:**
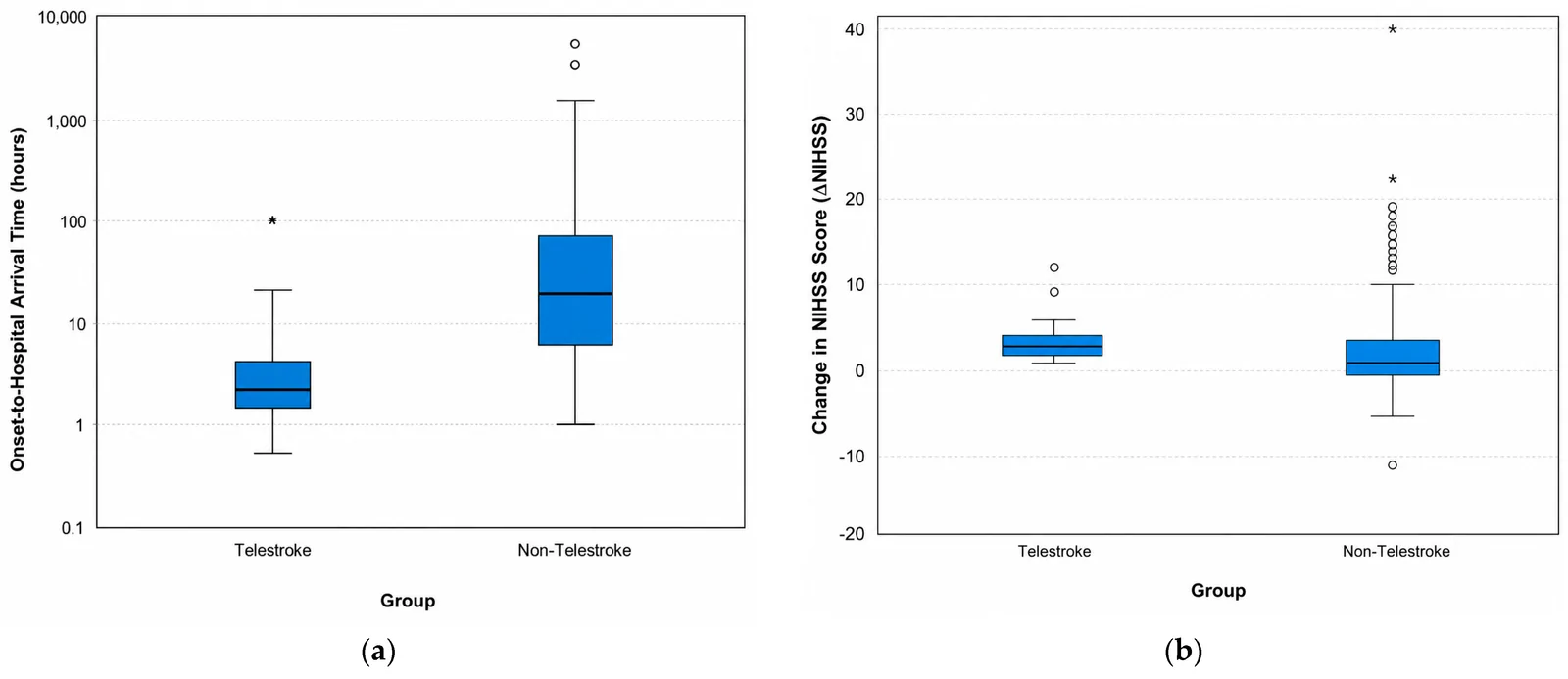
Boxplot Distribution of Onset-to-Admission Time and ΔNIHSS among Acute Stroke Patients in the Telestroke and Non-Telestroke Groups. (**a**) Onset-to-admission time; (**b**) ΔNIHSS. Circles indicate outliers, and asterisks (*) indicate extreme outliers.

**Table 1:** List of *spoke* hospitals.

No.	Name of the Network/Referrer Hospital	Address	Distance	Travel Time
1	Ujung Berung Regional General Hospital	Jl. Rumah Sakit No. 22, Pakemitan, Kec. Cinambo, Kota Bandung, Jawa Barat 40294	12.5 km	40–50 m
2	Bandung Kiwari Regional General Hospital	Jl. Moch. Toha No. 369, Ciseureuh, Kec. Regol, Kota Bandung, Jawa Barat 40256	8.4 km	30–40 m
3	Sartika Asih Bhayangkara Level II Hospital	Jl. Moch. Toha No. 369, Ciseureuh, Kec. Regol, Kota Bandung, Jawa Barat 40256	9.3 km	30–50 m
4	dr. M. Salamun Air Force Level II Hospital	Jl. Ciumbuleuit No. 203, Ciumbuleuit, Kec. Cidadap, Kota Bandung, Jawa Barat 40142	4.1 km	13–30 m
5	Bandung Advent Hospital	Jl. Cihampelas No. 161, Cipaganti, Kecamatan Coblong, Kota Bandung, Jawa Barat 40131	1.1 km	5–10 m

**Table 2:** Demographic characteristics of Study Subjects in Acute Stroke Patients between Telestroke Group and Non-Telestroke Patients.

Variable	Telestroke *n* = 39	Non-Telestroke *n* = 228	*p*-Value
Age (years)	57.41 ± 13.21	57.64 ± 13.21	0.918 ^a^
Gender (Male)	24 (61.5%)	116 (50.9%)	0.218 ^b^
Risk factors			
Hypertension	31 (79.5%)	209 (91.7%)	0.038 ^c^*
Diabetes Mellitus	12 (30.8%)	46 (20.2%)	0.138 ^b^
Prediabetes	0 (0.0%)	1 (0.4%)	1.000 ^c^
Smoking	8 (20.5%)	50 (21.9%)	1.000 ^c^
Previous strokes	3 (7.7%)	98 (43.0%)	<0.001 ^c^*
Dyslipidemia	10 (25.6%)	160 (70.2%)	<0.001 ^c^*
Hyperurisemia	4 (10.3%)	33 (14.5%)	0.481 ^b^
Atrial Fibrillation	3 (7.7%)	19 (8.3%)	1.000 ^c^
Rheumatic Heart Disease	1 (2.6%)	0 (0.0%)	0.146 ^c^
Chronic Kidney Disease	1 (2.6%)	30 (13.2%)	0.060 ^c^
Obstructive Sleep Apnea	1 (2.6%)	22 (9.6%)	0.217 ^c^
Polycythemia vera	1 (2.6%)	1 (0.4%)	0.271 ^c^
Hypercoagulability	1 (2.6%)	10 (4.4%)	1.000 ^c^

Note: Analysis was performed using ^a^ Unpaired *t*-test, ^b^ Chi-square, ^c^ Fisher’s exact test; * *p* < 0.05.

**Table 3:** Differences in Onset-to-Admission Time and ΔNIHSS among Acute Stroke Patients in the Telestroke and Non-Telestroke Groups.

Variable	Telestroke = 39	Non-Telestroke = 228	*p*-Value
Onset-to-admission time, median (IQR), h	3.5 (2.5–6.0)	20.0 (7.0–72.0)	<0.001 *
Onset-to-admission category, *n* (%)			<0.001 *
≤4.5 h	23 (59.0)	19 (8.3)	
>4.5 h	16 (41.0)	209 (91.7)	
Admission NIHSS, median (IQR)	7 (4–13)	7 (4–11)	0.478 ^a^
Discharge NIHSS, median (IQR)	4 (2–9)	4 (3–7)	0.652 ^a^
ΔNIHSS, median (IQR)	3 (2–5)	1 (0–4)	<0.001 *
ΔNIHSS, range	1–11	−11–40	
Direction of NIHSS change, *n* (%)			<0.001 *
Worsened	0 (0.0)	3 (1.3)	
Unchanged	0 (0.0)	99 (43.4)	
Improved	39 (100.0)	126 (55.3)	
NIHSS improvement ≥ 4 points, *n* (%)	14 (35.9)	65 (28.5)	0.350 ^b^
Length of hospital stay, median (IQR), days	5 (5–9)	6 (5–9)	0.425 ^a^
Length of hospital stay, range	2–35	0–41	

Note: ^a^ Mann–Whitney U test; ^b^ Chi-square test. * *p* < 0.05. Continuous variables are presented as median (IQR). LOS data were available for 20/39 patients in the Telestroke group and 223/228 patients in the Non-Telestroke group. ΔNIHSS was calculated as admission NIHSS minus discharge NIHSS; positive values indicate neurological improvement. NIHSS, National Institutes of Health Stroke Scale; IQR, interquartile range; LOS, length of hospital stay.

**Table 4:** Multivariable Logistic Regression for Onset-to-Admission ≤ 4.5 h.

Variable	aOR	95% CI	*p*-Value
Telestroke	11.20	4.16–30.11	<0.001 *
Age	1.02	0.98–1.05	0.361
Male sex	0.67	0.28–1.60	0.368
Admission NIHSS	1.02	0.94–1.11	0.678
Hypertension	1.98	0.53–7.45	0.312
Diabetes mellitus	0.86	0.33–2.26	0.755
Smoking	0.56	0.20–1.56	0.266
Previous stroke	5.18	1.61–16.71	0.006 *
Dyslipidemia	1.13	0.45–2.88	0.793
Atrial fibrillation	5.97	0.64–55.50	0.116
Chronic kidney disease	0.43	0.13–1.50	0.187

Note: Outcome: onset-to-admission ≤ 4.5 h. Model: Omnibus *p* < 0.001; Nagelkerke R^2^ = 0.376; Hosmer–Lemeshow *p* = 0.740. aOR, adjusted odds ratio; CI, confidence interval; NIHSS, National Institutes of Health Stroke Scale. * *p* < 0.05.

**Table 5:** Multivariable Logistic Regression for NIHSS Improvement ≥ 4 Points.

Variable	aOR	95% CI	*p*-Value
Telestroke	0.74	0.27–2.06	0.564
Age	1.00	0.97–1.03	0.972
Male sex	0.93	0.47–1.86	0.835
Admission NIHSS	1.35	1.25–1.46	<0.001 *
Hypertension	1.54	0.51–4.64	0.446
Diabetes mellitus	0.89	0.39–2.02	0.782
Smoking	0.99	0.41–2.37	0.977
Previous stroke	2.59	1.21–5.54	0.014 *
Dyslipidemia	1.29	0.63–2.66	0.492
Atrial fibrillation	0.80	0.26–2.46	0.698
Chronic kidney disease	1.18	0.40–3.52	0.761

Note: Outcome: NIHSS improvement ≥ 4 points. Model: Omnibus *p* < 0.001; Nagelkerke R^2^ = 0.403; Hosmer–Lemeshow *p* = 0.274. aOR, adjusted odds ratio; CI, confidence interval; NIHSS, National Institutes of Health Stroke Scale. * *p* < 0.05.

**Table 6:** Summary of the Propensity Score Matching Process.

Parameter	*n*	%
Initial Telestroke patients	39	100.0
Telestroke patients matched to 3 controls	22	56.4
Telestroke patients matched to 2 controls	2	5.1
Telestroke patients matched to 1 control	4	10.3
Telestroke patients without a match	11	28.2
Selected Non-Telestroke controls	74	—
Final matched sample	102	—

Note: Matching ratio up to 1:3, without replacement; caliper = 0.370 on the logit scale of the propensity score.

**Table 7:** Baseline Characteristics After Propensity Score Matching.

Variable	Telestroke = 28	Non-Telestroke = 74	SMD
Age, mean ± SD	56.93 ± 13.40	55.64 ± 13.48	0.096
Admission NIHSS, mean ± SD	7.64 ± 4.57	8.53 ± 4.55	−0.195
Male sex, *n* (%)	14 (50.0)	45 (60.8)	−0.219
Hypertension, *n* (%)	23 (82.1)	61 (82.4)	−0.008
Diabetes mellitus, *n* (%)	6 (21.4)	15 (20.3)	0.029
Smoking, *n* (%)	7 (25.0)	19 (25.7)	−0.016
Previous stroke, *n* (%)	3 (10.7)	9 (12.2)	−0.046
Dyslipidemia, *n* (%)	10 (35.7)	33 (44.6)	−0.182
Atrial fibrillation, *n* (%)	3 (10.7)	5 (6.8)	0.141
Chronic kidney disease, *n* (%)	1 (3.6)	4 (5.4)	−0.089

Note: SMD = standardized mean difference. Generally, |SMD| < 0.10 indicates good balance; values of 0.10–0.20 indicate mild-to-moderate residual imbalance.

**Table 8:** Clinical Outcomes after Propensity Score Matching.

Outcome	Telestroke = 28	Non-Telestroke = 74	*p*-Value
Onset ≤ 4.5 h, *n* (%)	16 (57.1)	7 (9.5)	<0.001 *
Thrombolysis, *n* (%)	5 (17.9)	3 (4.1)	0.034 *^a^
ΔNIHSS, median (IQR)	2 (2–3.75)	2 (0–5.25)	0.303
NIHSS improvement ≥ 4 points, *n* (%)	7 (25.0)	28 (37.8)	0.223
Length of stay, valid *n*	14	70	0.163

Note: ^a^ a Fisher’s exact test was used for thrombolysis because an expected cell count was <5. * *p* < 0.05. NIHSS, National Institutes of Health Stroke Scale; IQR, interquartile range.

**Table 9:** Logistic Regression Analysis after Propensity Score Matching.

Model/Variable	aOR	95% CI	*p*-Value
Onset-to-admission ≤ 4.5 h: Telestroke	13.53	4.48–40.86	<0.001 *
Onset-to-admission ≤ 4.5 h: Admission NIHSS	1.03	0.91–1.16	0.682
Onset-to-admission ≤ 4.5 h: Age (per year)	0.99	0.95–1.03	0.527
NIHSS improvement ≥ 4 points: Telestroke	0.61	0.18–2.03	0.419
NIHSS improvement ≥ 4 points: Admission NIHSS	1.39	1.22–1.59	<0.001 *
NIHSS improvement ≥ 4 points: Age (per year)	0.98	0.95–1.02	0.368

Note: Model for onset-to-admission ≤ 4.5 h: Nagelkerke R^2^ = 0.330; Hosmer–Lemeshow *p* = 0.822. Model for NIHSS improvement ≥ 4 points: Nagelkerke R^2^ = 0.419; Hosmer–Lemeshow *p* = 0.381. aOR, adjusted odds ratio; CI, confidence interval; NIHSS, National Institutes of Health Stroke Scale. * *p* < 0.05.

## Data Availability

The data presented in this study are available on request from the corresponding author. The data are not publicly available due to ethical restrictions.
